# Bone Marrow-Derived HipOP Cell Population Is Markedly Enriched in Osteoprogenitors

**DOI:** 10.3390/ijms130810229

**Published:** 2012-08-16

**Authors:** Shousaku Itoh, Kenta Matsushita, Shun Ikeda, Yumiko Yamamoto, Yukako Yamauchi, Seisuke Yoshioka, Reiko Yamamoto, Shigeyuki Ebisu, Mikako Hayashi, Jane E. Aubin

**Affiliations:** 1 Department of Restorative Dentistry and Endodontology, Osaka University Graduate School of Dentistry, 1-8, Yamada-oka Suita, Osaka 565-0871, Japan; E-Mails: m-kenta@dent.osaka-u.ac.jp (K.M.); ikeda@dent.osaka-u.ac.jp (S.I.); nagareda@dent.osaka-u.ac.jp (Y.Y.); yamauchi@dent.osaka-u.ac.jp (Y.Y.); yoshioka@dent.osaka-u.ac.jp (S.Y.); reiko-s@dent.osaka-u.ac.jp (R.Y.); ebisu@dent.osaka-u.ac.jp (S.E.); mikarin@dent.osaka-u.ac.jp (M.H.); 2 Department of Molecular Genetics, Faculty of Medicine, University of Toronto, Room 4245, Medical Sciences Building, 1 King’s College Circle, Toronto, ON M5S 1A8, Canada; E-Mail: jane.aubin@utoronto.ca

**Keywords:** osteoprogenitor, bone marrow stromal cell, mesenchymal stem cell, transplantation, tissue reconstruction

## Abstract

We recently succeeded in purifying a novel multipotential progenitor or stem cell population from bone marrow stromal cells (BMSCs). This population exhibited a very high frequency of colony forming units-osteoblast (CFU-O; 100 times higher than in BMSCs) and high expression levels of osteoblast differentiation markers. Furthermore, large masses of mineralized tissue were observed in *in vivo* transplants with this new population, designated highly purified osteoprogenitors (HipOPs). We now report the detailed presence and localization of HipOPs and recipient cells in transplants, and demonstrate that there is a strong relationship between the mineralized tissue volume formed and the transplanted number of HipOPs.

## 1. Introduction

Bone marrow harbors both an endosteal/osteoblastic niche and a vascular/sinusoidal niche for hematopoietic stem cells [1–3]. At least some cells within bone marrow stromal populations comprising the endosteal/osteoblastic niche, as established originally by Freidenstein [4], are multipotential and can differentiate *in vitro* into osteoblasts, chondrocytes, adipocytes and myoblasts, which has led to the population being designated mesenchymal stem cells, multipotential marrow stromal cells (MSCs) or skeletal stem cells (SSCs) amongst other names [4–9]. MSCs from human and rat bone marrow have been the most extensively characterized, because they are relatively easy to isolate by their phenotype of adherence to plastic and extensive expansion capacity in culture. In contrast, murine MSCs are far more difficult to isolate from bone marrow and to expand in culture [10], at least in part due to significant contamination by hematopoietic cells that are also directly adherent to plastic and bind to bone marrow stromal cells via adhesion molecules, cytokine receptors, and extracellular matrix proteins [11–13].

Recently, we reported a novel method for significant enrichment of MSCs from murine bone marrow using a magnetic micro-beads technique [14]. The enriched population is multipotential, with a very high frequency of colony forming units-osteoblast (CFU-O) and high expression levels of osteoblast differentiation markers in the fractionated compared to the unfractionated population, indicating significant enrichment of osteoprogenitor cells (highly purified osteoprogenitors or HipOPs), amongst other mesenchymal progenitors. To address the *in vivo* differentiation potential, we transplanted HipOPs on collagen sponges into immunodeficient mice and found them to be significantly enriched in cells with a high potential for reconstitution of the skeletal system *in vivo*. On transplantation, the progenitor-enriched fraction regenerates a bone organ structure comprising multiple lineages: osteoblasts, osteocytes, osteoclasts, sinusoidal and bone marrow cells. Thus, the HipOP fraction has potential for reconstruction of bone and the bone marrow microenvironments.

In this report, we address the detailed localization of HipOPs (donor cells) versus recipient cells in transplants and how the transplanted cell number relates to the total volume of mineralized tissue produced by HipOPs.

## 2. Results

### 2.1. Donor Cells Generate Multiple Lineages Throughout the Transplants

**Figure 1:**
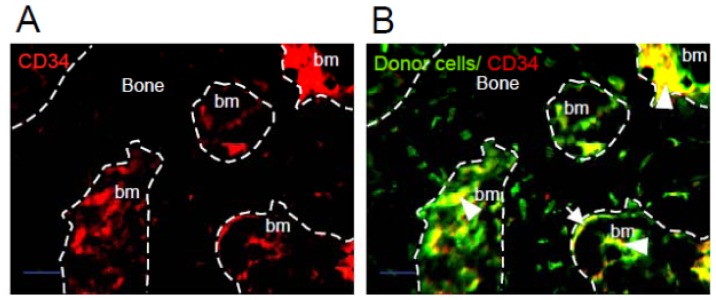
Donor highly purified osteoprogenitor (HipOP) cells in the transplant. HipOPs prepared from YFP transgenic mice {129-Tg(ACTB-EYFP)7AC5Nagy/J} were transplanted into nude mice. The transplants were recovered at 8 weeks after transplantation. Decalcified transplants were frozen in ornithine carbamoyltransferase compound with liquid nitrogen. Six micron sections were stained with anti-CD34 antibody (Red) and re-stained with Hoechst 33342 (Blue). CD34-positive donor cells were seen throughout the transplants, including in bone marrow (arrow head) and lining bone surfaces (arrow), ×100, bars = 50 μm. bm, bone marrow area.

**Figure 2:**
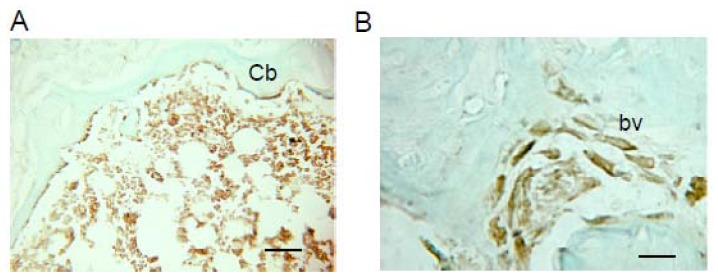
Histology of transplants of HipOPs harvested at 8 weeks. Frozen sections of transplants of HipOPs were stained with anti-H-2L^q^ Abs and re-stained with fast green. bv, blood vessel; Cb, cortical bone. (**a**) ×100, bar=50 μm; (**b**) ×400, bar = 30 μm.

When 1.5 × 10^6^ HipOPs in collagen sponges were transplanted subcutaneously into immunodeficient mice (Crlj:CD1-*Foxn1^nu^*), and analyzed at 8 weeks, bone-like tissue formed in the transplants, with an exterior of cortical bone and an interior cavity of trabecular bone structures with osteoblasts, osteocytes and bone marrow cells, and adipose tissue [14]. To confirm the presence and localization of donor cells, HipOPs prepared from yellow fluorescent protein (YFP) transgenic mice {129-Tg(ACTB-EYFP)7AC5Nagy/J} were transplanted in collagen sponges into nude mice. YFP positive cells (green) were observed throughout the bone-like tissue structure (Figure 1b). CD34 positive cells (red) were mainly localized in the bone marrow area (Figure 1a). YFP and CD34 double-positive cells (yellow) were observed in the bone marrow area, surrounding blood vessels (Figure 1b; arrow head) and on the bone surfaces (Figure 1b; arrow). HLA typing with H-2L^q^ antibodies showed that host or recipient cells (H-2L^q^: Crlj:CD1-*Foxn1^nu^* major histocompatibility complex) were also present throughout the structures, with the exception that no host osteocytes were seen (Figure 2a,b).

### 2.2. The Volume of Mineralized Tissue Produced Is Dependent on Transplanted by HipOP Cell Number

**Figure 3:**
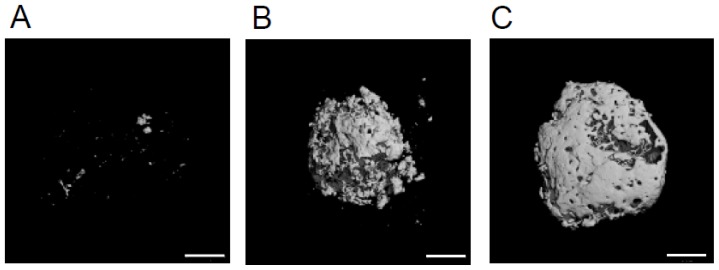
MicroCT 3D reconstructions of typical transplants of HipOPs at 8 weeks after transplantation. (**a**) 2.5 × 10^5^ transplanted cells; (**b**) 1.0 × 10^6^ transplanted cells; (**c**) 2.0 × 10^6^ transplanted cells, bars = 1 mm.

**Figure 4:**
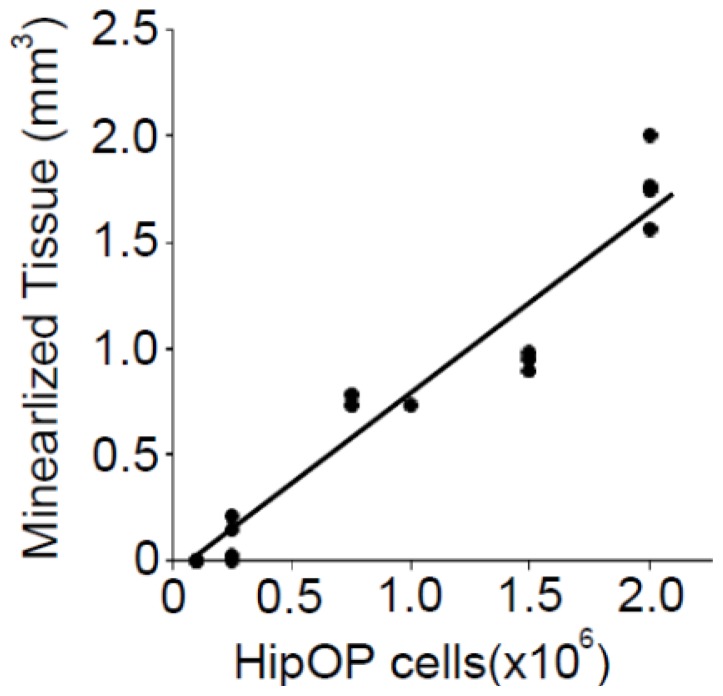
The relationship between the total mineralized tissue volume produced by HipOPs and the transplanted number of HipOPs.

When a small number (2 × 10^5^ cells) of HipOPs in collagen sponges was transplanted subcutaneously into immunodeficient mice (Crlj:CD1-*Foxn1^nu^*), and analyzed at 8 weeks, microCT photographs revealed that only a few small pieces of mineralized tissue were observed in transplants (Figure 3a). When a larger number (1 × 10^6^ cells; Figure 3b or 2 × 10^6^; Figure 3c) of HipOPs were transplanted, more mineralized tissue formed. Quantitative analysis revealed a linear relationship (*r* = 0.97) between the volume of mineralized tissue formed versus transplanted HipOP number (Figure 4). It is also worth noting that the amount of bone formed is saturable, *i.e.*, adding more HipOP cells (2.5 × 10^6^ and 3 × 10^6^ cells/sponge; data not shown) does not yield more bone.

## 3. Discussion

Recently, we succeeded in purifying a novel mesenchymal stem cell population (HipOPs), which has a high ability to produce mineralized tissue *in vitro* and *in vivo* [14]. To confirm the presence of host or donor cells within the tissue reconstituted in transplants, we used immunohistochemistry to differentially expressed major histocompatibility antigens in host versus donor cells and observed H2L^q^-positive (Crlj: CD1-*Foxn1^nu^* major histocompatibility complex) recipient cells throughout the bone-like tissue formed in transplants, with the exception of osteocytes (Figure 2). Thus, while both host and donor cells are present throughout transplants, the presence of only donor osteocytes in the organ structure formed suggests that the initial donor-derived bone may be responsible for recruiting host cells of multiple lineages to the transplant.

In our previous study [14], we showed that ~18% of cells in the HipOP fraction of bone marrow stromal cells are CD34-positive by flow cytometry, and we speculated that they were hematopietic cells. We now show by immunohistochemistry that CD34-positive cells are localized throughout the tissue reconstituted by HipOPs in transplants including not only cells within the bone marrow but, unexpectedly, also in osteoblast-like and lining cells along the bone surface and in other areas, including around blood vessels. This suggests that CD34 expression appears not to be limited to hematopietic cells but rather is also expressed in other cell types, including cells along bone surfaces and elsewhere, indicating that it is not an unequivocal marker of either hematopoietic or mesenchymal progenitors including MSCs but requires other markers to be used concomitantly for cell identification in mouse.

The osteoprogenitor frequency in HipOPs is more than 100 times higher than in unfractionated BMSCs (1/1,000 versus 1/140,000) as assessed by limting dilution analyses *in vitro* [14]. Linear regression analysis of the microCT data of transplanted HipOPs showed that there is not just more bone, but a strong linear relationship between the mineralized tissue volume and the transplanted HipOP cell number. Thus, in spite of HipOPs not being completely pure or homogeneous, there is no missing or contaminating subpopulation limiting for bone formation on transplantation. However, it is also notable that the amount of bone formed is saturable, *i.e.*, there is a threshold beyond which adding more HipOP cells (2.5 × 10^6^ and 3 × 10^6^ cells/sponge) does not yield more bone and we conclude that 1.5–2.0 mm^3^ of mineralized tissue is maximal bone formation under the conditions employed here.

## 4. Experimental Section

### 4.1. Isolation of HipOPs

Femurs of C57BL/6J mice (4–6 week-old) were harvested under sterile conditions and immersed in α-minimum essential medium (α-MEM) with antibiotics. After removal of the femoral heads, the marrow was collected by flushing repeatedly through the shafts with a syringe containing α-MEM supplemented with antibiotics and 10% heat-inactivated fetal calf serum (FCS), and sieving the cell suspension to remove cell aggregates. Recovered cells were plated in α-MEM supplemented with antibiotics as above and 10% FCS. After 3 days, nonadherent cells were removed by washing 3 times with PBS. Approximately 2 weeks after seeding, when the adherent cells had expanded to ~80% subconfluence, cells were detached with trypsin-EDTA solution (0.2% trypsin, 1mM EDTA). HipOPs were purified by negative sorting using anti-CD5, CD45, CD11b, Gr-1, 7-4, Ter-119 and CD45R conjugated magnetic beads (Miltenyi Biotec: Auburn, CA, USA) [14].

### 4.2. *In Vivo* Transplantation

*In vivo* transplantation was performed as reported [9,15]. HipOPs [C57BL/6J or YFP transgenic mice {129-Tg(ACTB-EYFP)7AC5Nagy/J}] were suspended in α-MEM containing 20% FCS. To load sponges (Gelfoam; Pfizer) with cells, the sponges were placed into the cell suspension and incubated for 90 min at 37%. The sponges were transplanted subcutaneously into 8- to 15-week old female Crlj:CD1-*Foxn1^nu^* mice. The transplants were recovered at 8 weeks after transplantation, fixed in PLP fixative (containing 4% paraformaldehyde) for 6 h at 4 °C, and decalcified with 15% EDTA for 1 week at 4 °C. Decalcified transplants were frozen in ornithine carbamoyltransferase compound with liquid nitrogen. Six micron sections were stained with anti-CD34 antibody (Biolegend) and Hoechst 33342 (Calbiochem). H-2L^q^ positive cells were detected by staining sections with biotinylated anti-H-2L^q^ antibody and a Vectastain Elite ABC kit [16]. Experimentation was conducted in accordance with the Japanese Guide for the Care and Use of Laboratory Animals and approved by the Animal Care Committee at the Osaka University.

### 4.3. Micro CT

The transplants were recovered at 8 weeks after transplantation. A detailed qualitative and quantitative 3D evaluation of the whole transplants was performed using a Scanco μCT40 scanner with 12 μm resolution (SCANCO Medical AG). A fixed threshold was applied to assess mineralized bone on the grey scale images. The threshold was chosen using 2D evaluation of several slices in the transverse anatomical plane so that mineralized tissue were identified while surrounding soft tissue was excluded. An average threshold of 265 was optimal and used uniformly for all samples. The total mineralized tissue volume was used for statistical analysis.

### 4.4. Statistical Analysis

Comparisons between means were made by using a Student’s *t*-test, and differences between means were considered significant when *P*-values were less than 0.05.

## 5. Conclusions

We have purified a novel bone marrow-derived population that manifests robust enrichment for cells with high potential for reconstitution of a multi-lineage bone organ structure *in vivo*. The data suggest that further development of the purification methods and culture system, including for example development of a FCS-free culture system for cell expansion, may position this novel population for regenerative medicine applications, including those necessary in certain orthopedic or dental applications.
